# To further incorporate computer-aided designs to improve preoperative planning in total hip arthroplasty: a cohort study

**DOI:** 10.3389/fsurg.2024.1345261

**Published:** 2024-07-08

**Authors:** Kai Cheng, Haotian Zhu, Yuanhao Peng, Han Yan, Xinghua Wen, Zixuan Cheng, Huanwen Ding

**Affiliations:** ^1^ Department of Orthopedics, Guangzhou First People’s Hospital, Guangzhou, China; ^2^ School of Medicine, South China University of Technology, Guangzhou, China; ^3^ School of Biomedical Sciences and Engineering, South China University of Technology, Guangzhou, China; ^4^ Department of Radiology, Guangzhou First People’s Hospital, Guangzhou, China

**Keywords:** total hip arthroplasty, computer-aided design, preoperative planning, personalized therapy, preoperative simulation

## Abstract

**Background:**

Hip replacement surgeries are increasing in demand, requiring rigorous improvements to a mature surgical protocol. Postoperative patient dissatisfaction mainly stems from postoperative complications resulting from the inappropriate selection of prostheses to meet the needs of each patient. This results in prosthesis loosening, hospital-related fractures, and postoperative complex pain, which can all be attributed to inappropriate sizing. In this study, we aimed to further explore the intraoperative and postoperative benefits of incorporating computer-aided design (CAD) in preoperative planning for total hip arthroplasty (THA).

**Methods:**

A total of 62 patients requiring total hip replacement surgery from January 2021 to December 2021 were collected and randomly divided into a preoperative computer-aided simulated group and a conventional x-ray interpretation group. The accuracy of implant size selection (femoral and acetabular implant) between the preoperative planning and surgical procedure of the two groups was compared. Patient parameters, perioperative Harris hip scores, operative time (skin-to-skin time), surgical blood loss, and postoperative hospital stay were recorded, and the differences between the two groups were statistically compared using a single sample *t*-test.

**Results:**

All patients in the study were successfully operated on and achieved good postoperative functional recovery. With CAD, the selection of the most suitable-sized prosthesis was significantly more accurate compared to the control group (accuracy of the acetabular component between the CAD/control: 80.6%/61.3%, and accuracy of the femoral component: 83.9%/67.7%). Intraoperative blood loss (177.4/231.0 ml, *P* = 0.002), operation time (84.2 ± 19.8 min/100.3 ± 25.9 min, *P* = 0.008), duration of hospital stay (6.5 ± 3/9.1 ± 3.9 days, *P* = 0.003), and postoperative Harris hip score (81.9 ± 6.5/74.7 ± 11.1, *P* = 0.003) were compared to the control group and showed statistical significance.

**Conclusion:**

Incorporating CAD into the preoperative planning of total hip arthroplasty can effectively guide the selection of the most suitable-sized prosthesis, reduce intraoperative blood loss, and promote short-term functional recovery after THA.

## Background

With an aging society, the incidence of geriatric diseases such as osteoarthritis of the hip and osteonecrosis of the femoral head requiring end-stage surgical treatment like total hip replacement (THA) is gradually increasing (1). Numerous factors influence postoperative functional recovery, including living habits of patients, underlying diseases, postoperative planning, surgical approach, the type and model of the implant prosthesis used, and postoperative functional rehabilitation (2–4). Among these factors, proper preoperative planning critically affects postoperative results. Accurate selection of the appropriate prosthetic size implanted during surgery greatly affects postoperative results and functional recovery of the operated limb. It is still common practice to rely on standardized preoperative plain radiographs as a reference, although this approach has limitations (5–7). Inaccurate measurements affect preoperative planning which consequently leads to prolonged surgical duration due to intraoperative testing of numerous components. Inaccurate prosthesis selection and placement also increases the risk of intraoperative fractures, postoperative lower limb discrepancies, implant dislocation, and implant loosening. Such complications delay the overall recovery process, increase the risk of early hip revision, further increase hospitalization costs, and negatively impact quality of life (8–10).

Advances in medical engineering have made numerous contributions to overcoming the limitations of radiograph-based preoperative planning. In a study by Petretta et al., preoperative planning based on acetate templating on digital images provided superiorly accurate measurement of the required prosthetic size (11). Chen et al. and Di Laura et al. utilized artificial intelligence in their studies to develop algorithms that automatically identify sizes of the prosthesis components required and select them appropriately according to patient demographics (12, 13). Incorporating CAD during preoperative planning is also beneficial for the optimal placement of the acetabular prosthesis, including determining implant anteversion and center of rotation, which provides better implant stability. This approach decreases hospital expenses by omitting the need for an acetabular screw, thereby reducing the economic burden of THA (14). This was previously studied by Winter et al., who showed that 3D templating provides favorable guidance for acetabular selection and placement in hip revision surgeries (15). Although these strategies have shown potential, they have yet to reach common usage in the clinical setting due to software developmental costs, the complexity of the procedures involved, and unstable results. Further studies are thus required to explore a more economical and efficient solution.

Presently, preoperative planning utilizing computer-aided design (CAD) based on DICOM data derived from CT scans has been widely used in the clinical setting. The use of engineering software allows for the three-dimensional model construction of human organs, reaching high levels of precise measurements and surgical simulations. Studies have demonstrated the feasibility and efficiency of CAD in preoperative planning, intraoperative assistance, and postoperative evaluation (16–18). However, studies utilizing CAD in the preoperative planning of THA to assist in selecting a suitable prosthesis have yet to reach clinical popularity. Our study is dedicated to (i) utilizing CAD surgical simulations of THA to assist in selecting the most suitable-sized prosthesis; (ii) improving the accuracy and personalize THA preoperative planning; and (iii) improving postoperative rehabilitation and surgical outcomes in THA patients through better personalized CAD-guided care.

## Methods

### Patients

From January 2021 to December 2021, patients requiring total hip replacement surgery at our hospital were included in our study. The inclusion criteria were (1) patients undergoing primary total hip arthroplasty and (2) patients diagnosed with a femoral neck fracture, osteonecrosis of the femoral head, and hip osteoarthritis. Patients were excluded from the study if (1) undergoing revision hip arthroplasty, (2) diagnosed with development dysplasia of the hip (DDH), and (3) having life-threatening comorbidities such as respiratory and cardiac insufficiency. This study was approved by the IRB Ethics Institute of Guangzhou First People's Hospital (K-2018-137-04). Patient-specific information is given in Table 1.

**Table 1:** Basic information of enrolled patients, including patient age, sex, number of operated left or right limbs, and preoperative Harris hip scores (*T = */).

	Age (years)	Sex (M/F)	Operated limb (L/R)	Preoperative Harris hip score
CAD group	60.5 ± 13.2	16/15	12/19	48.1 ± 9.7
Control group	60.2 ± 12.5	14/17	17/14	49.2 ± 4.7
Test value	0.079	—	—	−0.597
*P*-value	0.93	—	—	0.55

### Preoperative planning

**Figure 1:**
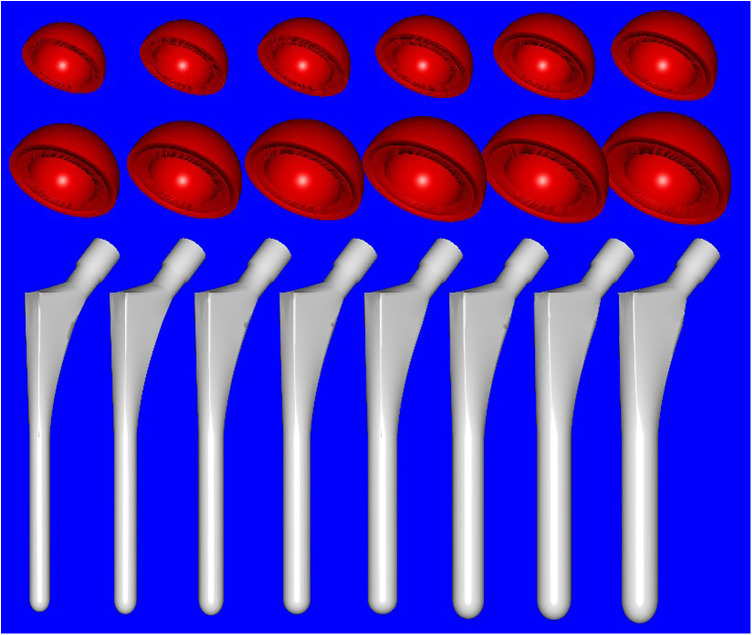
Full digital model of hip prosthesis used in the study (Smith and Nephew, London, UK): acetabular cup (red, from left to right) measuring from 36 to 58 mm in diameter with an increment of 2 mm for each size and femoral stem (white, from left to right) measuring from 7 to 14 mm in diameter of the distal stalk with 1 mm increment for each size.

All patients in our study underwent preoperative anteroposterior (AP)-lateral hip radiography and pelvic CT scanning. A 64-slice spiral CT was used to conduct thin-sliced plain scans of the affected hip joint, with a layer distance and thickness of 0.625 mm. The DICOM data were then imported into medical image processing software Mimics20.0 (Materialize Software, Leuven, Belgium). A three-dimensional anatomical model of the hip joint was generated and digitally processed into an STL format. The STL data model was then imported into reverse engineering software Imageware13.0 (UGS Corporation, Plano, TX, USA), and the anatomical structures involved in THA preoperative planning (femoral head spherical boundaries, femoral head and neck axis, femoral shaft axis) were measured. The hip replacement systems utilized in our study were obtained from a single product line [Smith and Nephew (SN), London, United Kingdom]. The prosthetic parameters of the entire SN product line (including the acetabular and femoral component) were digitized using a laser scanner and processed using Imageware to obtain 3D STL data (Figure 1). This included diameter, depth, femoral stalk length, width, angular structure, and curvature, with a degree of error up to 0.05 mm, which can be directly utilized for preoperative planning and surgical simulations.

**Figure 2:**
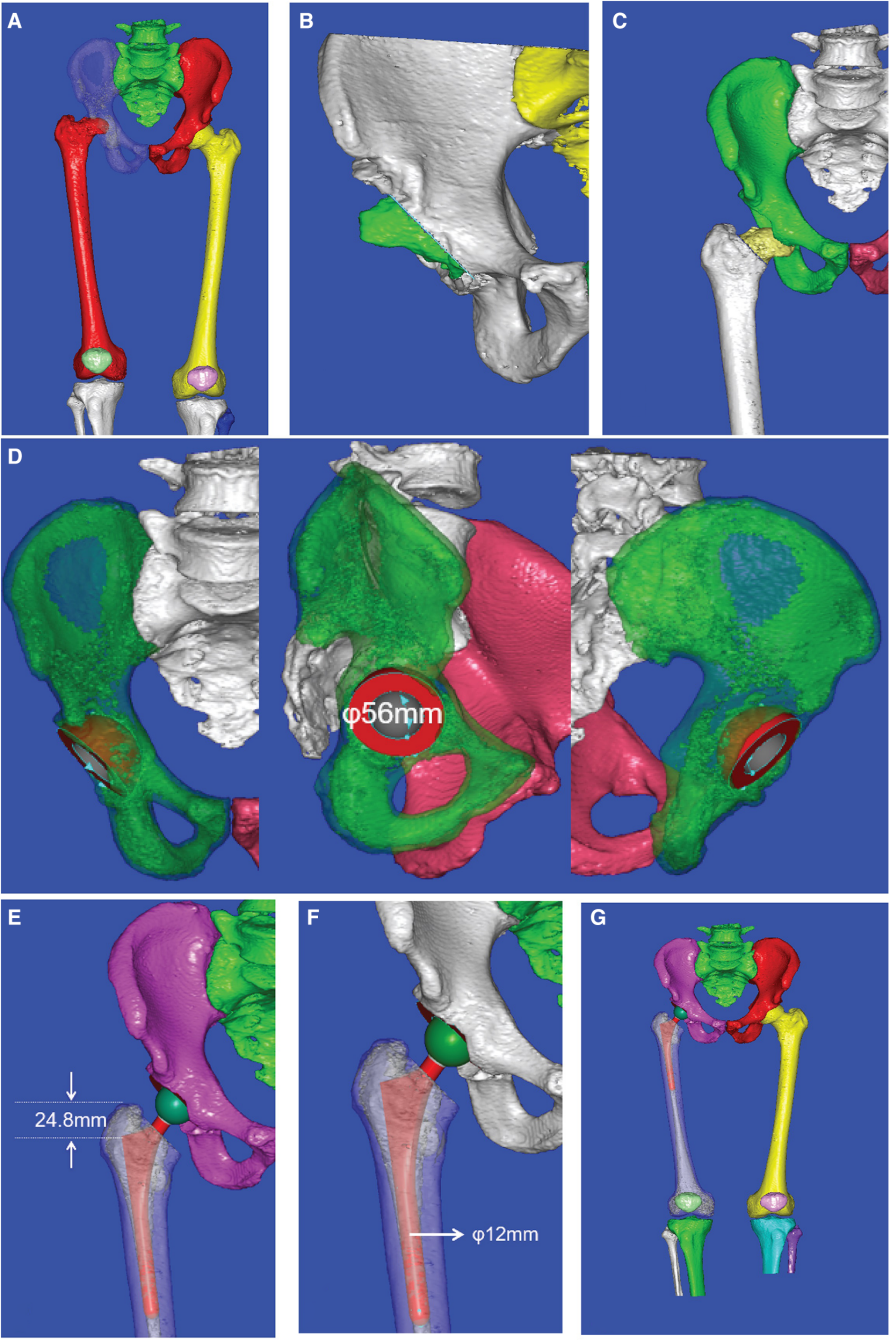
Preoperative CAD planning process: (**A**) 3D reconstruction of the hip joint, (**B**) simulation of the limb after periacetabular osteophyte removal, (**C**) determination of the femoral neck osteotomy position, (**D**) selection of acetabular cup size, (**E**) determination of the depth for femoral stem insertion, (**F**) selection of femoral stem size, and (**G**) simulation of the hip structure after THA.

Intraoperative acetabular cup implantation was simulated in the software with a standardized abduction angle of 40° and an anteversion angle of 15°; the femoral prosthesis was inserted along the medullary canal. The selection of the most suitable acetabular component size was based on a standardized total hip arthroplasty templating criterion, ensuring that the outer metallic curvature of the acetabular cup made contact with the acetabular subchondral bone and the inferior border of the metallic cup lay within and parallel to the transverse ligament. The most suitable femoral implant size was selected according to the intramedullary diameter of the proximal femur. The proximal stem was positioned parallel to the site of femoral neck osteotomy and can be evaluated intraoperatively by hand-rotating the femoral stem after placement to assess for axial stability. The overall preoperative planning process in our study is shown in Figure 2.

### Surgical procedure

All patients underwent a standard posterolateral approach for THA, performed by the same surgical team. After anesthesia induction, a curved 6–8 cm posterolateral incision was made over the greater trochanter. The soft tissue layer was dissected to expose the joint capsule, and the femoral head was then removed by osteotomy. Subsequently, the joint capsule was dissected to expose the medial side of the acetabulum. The cup implant and polyethylene lining were placed after grinding the acetabular soft tissue using an acetabular reamer. The soft tissue lining of the acetabular surface was removed, followed by femoral broaching to expose the femoral canal for insertion of the femoral prosthesis, completing the implantation of both femoral and acetabular components. All of the final sizes used were in accordance with the intraoperative findings, with preoperative sizing mainly used as a reference. After successful reduction, the exposed articular cavity was washed with saline, and 30 ml of cocktail solution was injected into the surrounding soft tissues. The hip capsule was then reconstructed, followed by repair of the posterior tissue envelope. Finally, the surgical incision was sutured and closed.

### Perioperative management and postoperative rehabilitation

Preoperative antibiotics were administered 30 min prior and 24 h after surgery to prevent postoperative infection. Low-molecular-weight heparin was administered starting from the second postoperative day to prevent deep vein thrombosis, and quadriceps exercises were encouraged for all patients as soon as possible. Standard postoperative radiographs were taken on the second postoperative day to evaluate the accuracy and stability of the implanted prosthetics. Some of the patients were further evaluated using a postoperative CT scan of the operated limb. Patients were followed up at 1, 3, and 6 months postoperatively. Prosthetic loosening and dislocation were confirmed using femoral x-rays during the follow-up period, and the Harris hip function score was evaluated at the final follow-up (6 months post-operation) to further evaluate hip function recovery.

### Statistical analysis

SPSS22.0 statistical software was used for all of our data analysis. Data measurements in our study were expressed as mean ± standard deviation. A record was made of the instances where the acetabular and femoral component sizes were selected according to the preoperative planning vs instances where they differed from preoperative planning. These values were compared and analyzed. The intraoperative time, blood loss, postoperative observation time, and postoperative Harris hip score of the two groups were analyzed by independent sample *t*-tests. All data with a *p*-value <0.05 indicated statistical difference.

## Results

### Patient parameters

A total of 62 patients were included, with 31 patients in the CAD simulation group and 31 patients in the traditional x-ray interpretation control group. There were no significant differences in gender, age, and preoperative Harris hip scores between the two groups (*P* > 0.05). Details of all the patients are presented in Table 1.

### Accuracy of implant sizing

**Figure 3:**
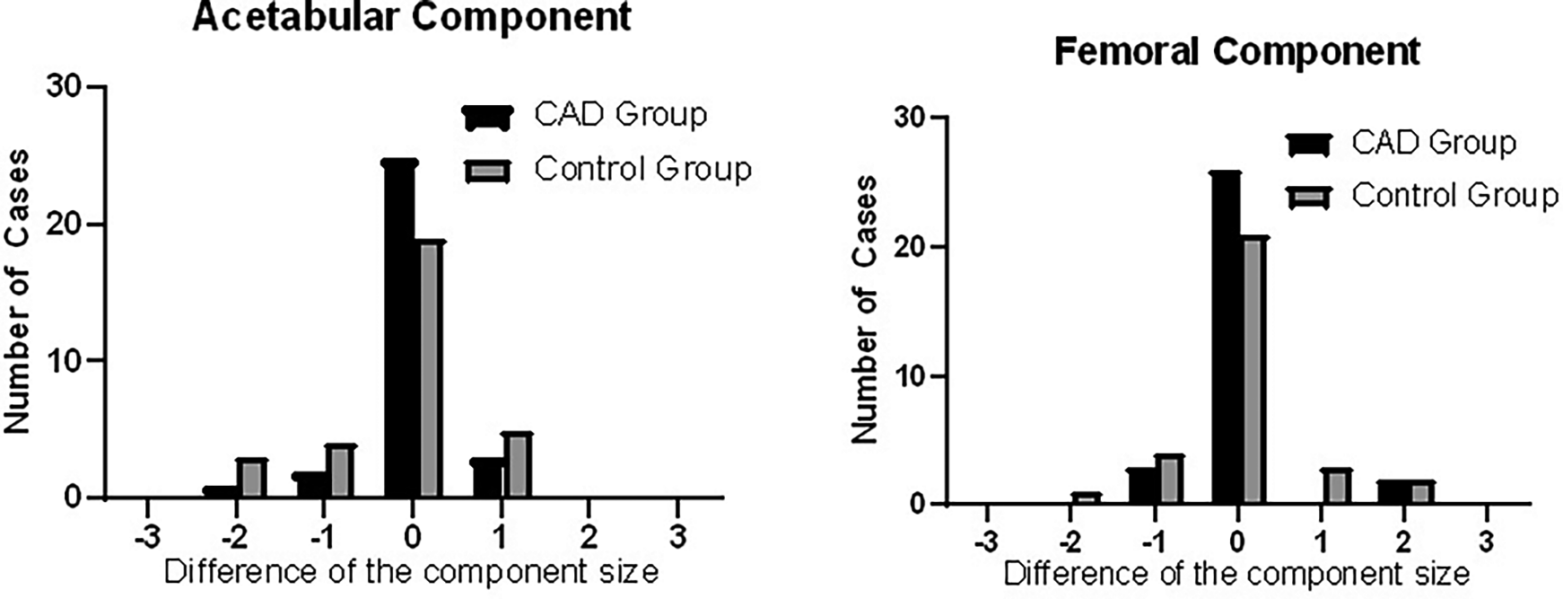
Histograms showing the difference in the intraoperatively used prosthesis size compared to the preoperative estimation through CAD preoperative planning. The prosthesis sizes are represented as “0” if the estimated size was accurate, “−” if the used prosthesis size was smaller than estimated, and “+” if the used prosthesis size was larger than estimated.

Data analysis of the comparison between the acetabular component selected during preoperative planning and the actual component used during the surgery showed accuracies of 80.6% and 61.3% and 83.9% and 67.7% for the femoral component between the CAD group and the control group, respectively. The number the implants used during surgery as compared to the preoperative planning of the two groups are presented in Figure 3 and Table 2.

**Table 2:** Numbers of correct and incorrect implant size prediction for the acetabular and femoral components in both groups.

	Acetabulum component		Femoral component	
	Accurate	Inaccurate	Accurate	Inaccurate
CAD group	25/31	6/31	26/31	5/31
Control group	19/31	12/31	21/31	10/31

### Surgical parameters

Intraoperative time, blood loss, duration of postoperative hospital stay, and final follow-up Harris hip score of the two groups were compared. The CAD group showed better results in all the recorded parameters (operation time, intraoperative blood loss, duration of postoperative hospital stay, and postoperative Harris hip score). The average operation time and intraoperative blood loss were found to be significantly lower in the CAD group than in the control group (operation time: 84.2 ± 19.8 vs. 100.3 ± 25.9 min, *P* = 0.008; blood loss: 177.4 ± 45.5 vs. 231.0 ± 76.7 ml, *P* = 0.002). The duration of postoperative hospital stay was also significantly lower in the CAD group than in the control group (6.5 ± 3.0 vs. 9.1 ± 3.9 days, *P* = 0.003). The Harris hip scores evaluated during follow-up at 6 months postoperatively were also significantly higher for the CAD group than for the control group (81.9 ± 6.5 and 74.7 ± 11.1, *P* = 0.003) (Table 3).

**Table 3:** Comparison of intraoperative and postoperative parameters between the two groups.

	Operation time (min)	Blood loss (ml)	Postoperative hospital stay time (days)	Postoperative Harris hip score
CAD group	84.2 ± 19.8	177.4 ± 45.5	6.5 ± 3.0	81.9 ± 6.5
Control group	100.3 ± 25.9	231.0 ± 76.7	9.1 ± 3.9	74.7 ± 11.1
Test value	−2.759	−3.347	−3.017	3.116
*P*-value	0.008	0.002	0.003	0.003

### Complications and postoperative rehabilitation

**Figure 4:**
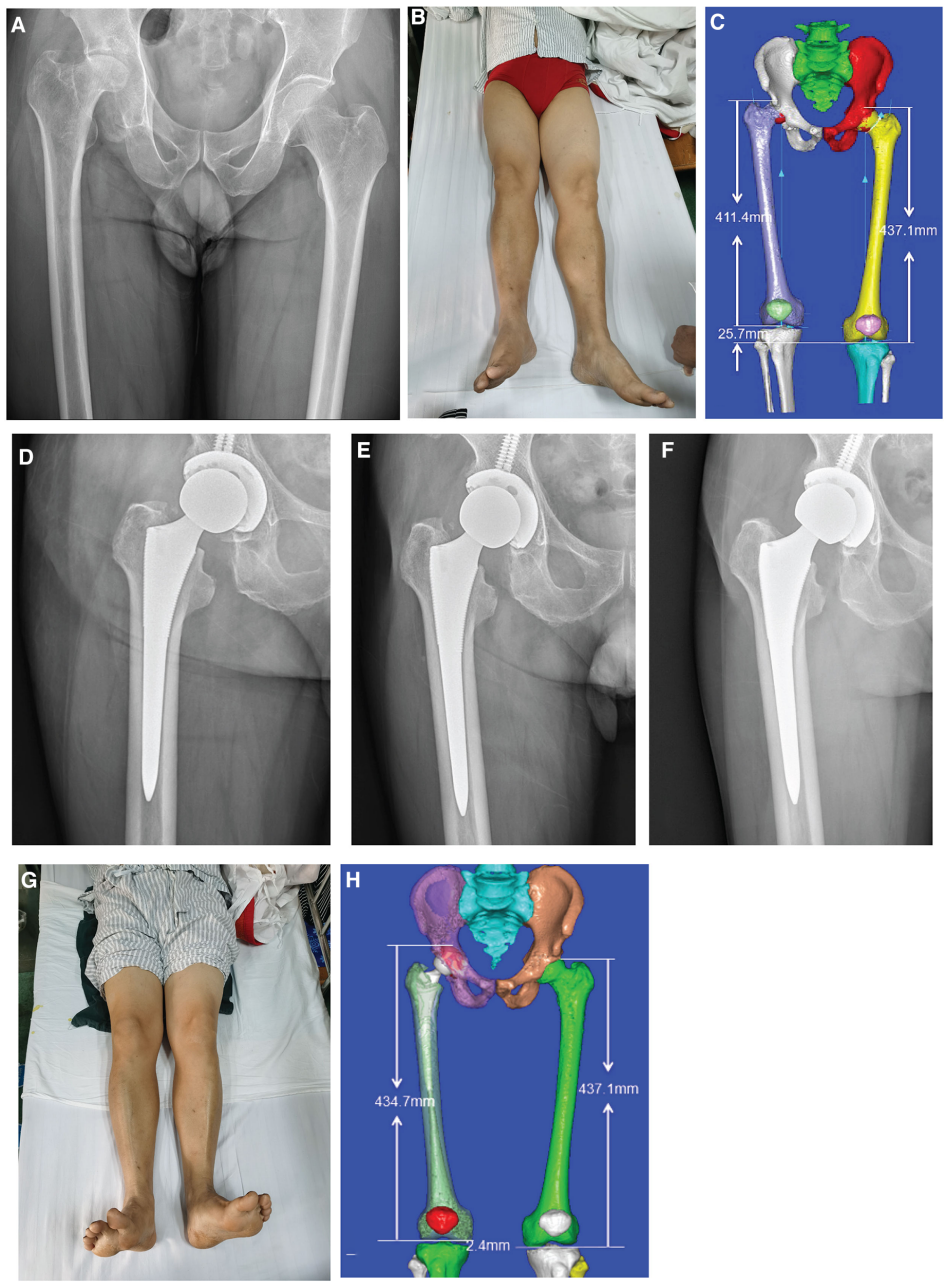
Typical case of CAD preoperative planning: (**A**) preoperative pelvic x-ray showing osteonecrosis of the femoral head (right side); (**B**) preoperative appearance of both lower limbs showing an obvious shortening of the right lower limb; (**C**) preoperative three-dimensional measurement of lower limb length difference; (**D–F**) follow-up radiographs at 1, 3, and 6 months postoperatively; (**G**) postoperative appearance of lower limbs; and (**H**) postoperative three-dimensional measurement of the lower limb length difference.

All patients underwent successful THA and were discharged after satisfactory wound healing without any incidence of surgical site infection during their hospital stay. There were no recorded incidences of acetabular penetration or proximal femoral fracture during prosthetic implantation. Patients achieved satisfactory joint stability and range of motion after surgery and were discharged without any incidence of surgical site infection. During discharge, all patients were asked to be followed up at 1, 3, and 6 months. Throughout the follow-up period, we did not encounter any incidence of infection, implant dislocation, periprosthetic fracture, or perioperative comorbidities (severe pneumonia, deep vein thrombosis, etc.). The stability of the implant was mainly evaluated by follow-up hip radiographs, which showed no evidence of loosening. Patients underwent physical examinations during their follow-ups to assess postoperative Harris hip scores. All patients in our study were able to return to regular daily activities without hip pain at 1–2 months after surgery. A sample case is presented in Figure 4.

## Discussion

### Limitations and prospects of further clinical application

We do acknowledge the limitations of our study, including the small population size and the lack of a multicenter large sample comparative study. In our study, we only included the different sizes from one series of prostheses and did not perform an analysis on the positioning of the prostheses during surgery. This may also lead to problems with postoperative rehabilitation in some patients. However, with CAD, the appropriate placement of the implant prostheses can be tailored for each patient with the usage of personalized surgical guide plates. Our future studies will aim to include the usage of surgical guide plates to further incorporate CAD in the proper placement of implant prostheses.

Personalized treatment is a current aim in the development of orthopedic surgical treatment because patients require specialized preoperative planning and surgical treatment plans unique to each individual to achieve an accurate diagnosis, treatment, and postoperative rehabilitation (19, 20). THA, as the end-stage therapy of choice for numerous hip diseases, is becoming a common surgical procedure with numerous strategies to improve surgical outcomes. Our study results show that implementing CAD in the preoperative planning of THA leads to better surgical outcomes compared to traditional radiograph assessment for implant sizing. This is reflected by a more accurate selection of the required implant size, resulting in a shorter surgical duration, reduced surgical blood loss, shorter postoperative hospital stay, and improved short-term postoperative functional recovery. The utilization of CAD in the preoperative planning of DDH, complex hip arthroplasties, and revision hip arthroplasties has been studied and shown to have evident benefits. Our study results show that CAD is also beneficial in the preoperative planning of regular total hip replacements.

### Improving preoperative planning in THA

Proper preoperative planning and the selection of suitable implant prostheses of both acetabular and femoral components used during surgery determine the surgical outcome and affect the postoperative quality of life. Inappropriate selection of an acetabular prosthesis that is too large for the acetabular rim leads to excessive degeneration of the polyethylene lining and increases the risk of pelvic wall penetration during surgery. This leads to implant instability and is one of the reasons for postoperative chronic pain. Conversely, selecting a cup implant that is too small leads to overall instability of the acetabular complex due to being unable to achieve a suitable press-fit, resulting in an inability to restore the physiological axis of the operated limb, further increasing the risk of wear and dislocation. Selecting an appropriate femoral implant size plays a vital role in the overall implant lifespan. Inappropriate selection of a femoral prosthesis that is too small for the intramedullary canal severely increases the risk of aseptic loosening (2, 10). Postoperative loss of trabecular bone volume causes the implant to lose the support of the surrounding tissue due to not having a press-fit relation with the surrounding bone tissue. On the other hand, selecting a femoral prosthesis that is too large directly increases the risk of periprosthetic fracture intraoperative and postoperatively (21–23). Therefore, appropriate preoperative planning and accurate intraoperative prosthesis selection are the first and most crucial steps in determining the success of total hip arthroplasty.

### Role of CAD in THA preoperative planning

The usage of CAD in our study allows for a 3D reconstruction of the operated area, providing an accurate understanding of the hip joint, including the severity of osteophyte growth surrounding the acetabular structures, the degree of degeneration of the acetabulum and femoral head, the accurate position of fractures and bony fragments, and the precise degree of fracture dislocation. For patients with femoral neck fractures, this offers guidance for determining the appropriate site of osteotomy. CAD mainly allows for the accurate measurement of acetabular margins, including both the diameter and depth in 3D, allowing for the most appropriate selection of a suitable prosthetic size. Preoperative surgical simulation by CAD allows for perioperative assessment of the operated limb, including length and axis changes, evaluation of the surgical plan, and early recognition and avoidance of the limitations and possible surgical complications. In our study, CAD greatly assisted in the preoperative planning for osteoporotic patients requiring THA. A 3D reconstruction of the affected limb allowed for a *better understan*ding of the hip and femoral bone mineral density, enabling proper avoidance of regions of vulnerability, thus preventing hospital-related injuries. Proper selection of prosthesis size also helps prevent prosthesis implant-related fractures, providing guidance as to whether the acetabular prosthesis further requires the usage of screws.

### CAD improves THA surgical parameters

In our study, we utilized CAD during preoperative planning to allow the surgeon to accurately predict the appropriate size of implant prostheses required. This allowed for a better selection of the size of the reamer and broach used instead of having to progressively increase the size of the instrument over a wide range. This contributes to effectively reducing the amount of time needed for reaming the soft tissue layer of the acetabulum and broaching the intramedullary canal of the femur, thus reducing the overall surgical time (which was recorded from making the surgical incision to suturing the surgical wound). The reduced amount of intraoperative trauma to surrounding tissue then reduces the overall amount of blood loss and the need for blood transfusions, effectively eradicating the risk associated with it. These factors promote patient postoperative rehabilitation and success to a certain degree. This is in accordance with Moyer et al., who reported that early postoperative rehabilitation relies on a precise surgical procedure (24). Our results show that the CAD group has a more accurate prediction of the acetabular prosthesis than the control group. Considering the postoperative evaluation, this means that there is a reduction in overall postoperative hospital stay and treatment costs. Postoperative follow-up also showed better results for the CAD group than for the control group, further proving the benefits of CAD in preoperative planning. In our study, we also recorded that the patients with an apparent preoperative leg length difference achieved simultaneous correction. Preoperative simulation, estimating the postoperative limb length difference after implanting the femoral prosthesis, allows for selecting a suitable femoral head component, which mainly relies on the subjective decision of each surgeon in the control group and would then be standardized through CAD simulation.

In our study, most of the elderly female patients require total hip replacement due to femoral neck fractures. The higher incidence of osteoporosis in this age group explains their susceptibility to fractures and requirement for additional attention in selecting appropriate implant prosthesis sizes to prevent pelvic wall penetration and femoral fractures during prosthesis implantation. Evidence of osteoporosis also provides a reminder of the necessity of osteoporotic treatment postoperatively. Consequently, the elderly male patients in our group require THA primarily due to osteonecrosis of the femoral head and their smoking and drinking habits. In this group of patients, the incidence of osteoporosis is relatively lower but mainly requires the cessation of their smoking and drinking habits postoperatively and postoperative vasoactive supportive treatment.

## Conclusion

Our results show that using CAD for preoperative planning improves the accuracy of the prosthesis selection to be used during surgery and provides a degree of benefit for postoperative rehabilitation. Standardizing CAD in the preoperative planning of primary total hip replacements benefits both the surgeon and the patient, which will also further benefit complex hip arthroplasties, including but not limited to DDH and revision arthroplasties.

## Data Availability

The raw data supporting the conclusions of this article will be made available by the authors, without undue reservation.
